# Large language models for drug discovery and development

**DOI:** 10.1016/j.patter.2025.101346

**Published:** 2025-09-02

**Authors:** Yizhen Zheng, Huan Yee Koh, Jiaxin Ju, Madeleine Yang, Lauren T. May, Geoffrey I. Webb, Li Li, Shirui Pan, George Church

**Affiliations:** 1 Department of Data Science and AI, Monash University, Melbourne, VIC, Australia; 2 Drug Discovery Biology, Monash Institute of Pharmaceutical Sciences, Monash University, Melbourne, VIC, Australia; 3 School of Information and Communication Technology, Griffith University, Gold Coast, QLD, Australia; 4 Department of Genetics, Blavatnik Institute, Harvard Medical School, Boston, MA, USA; 5 Wyss Institute for Biologically Inspired Engineering, Harvard University, Boston, MA, USA

**Keywords:** large language models, drug discovery, drug development

## Abstract

The integration of large language models (LLMs) into the drug discovery and development field marks a significant paradigm shift, offering novel methodologies for understanding disease mechanisms, facilitating *de novo* drug discovery, and optimizing clinical trial processes. This review highlights the expanding role of LLMs in revolutionizing various stages of the drug development pipeline. We investigate how these advanced computational models can uncover target-disease linkage, interpret complex biomedical data, enhance drug molecule design, predict drug efficacy and safety profiles, and facilitate clinical trial processes. In this paper, we aim to provide a comprehensive overview for researchers and practitioners in computational biology, pharmacology, and AI4Science by offering insights into the potential transformative impact of LLMs on drug discovery and development.

## The bigger picture

Developing new medicines is a notoriously long and expensive process, often taking over a decade and costing billions of dollars per drug. This lengthy timeline significantly delays patient access to life-changing treatments. Artificial intelligence, particularly large language models (LLMs) that can understand both scientific and human language well, offers a promising solution to this challenge. While initially designed to understand and generate human language, these LLMs are now being adapted to “understand” scientific data, including the complex language of DNA, proteins, and chemical structures.

This paper highlights how LLMs are becoming crucial tools at every stage of drug development. For instance, they can help researchers quickly pinpoint the biological causes of diseases, even suggesting new drug targets. LLMs can also design entirely new drug molecules from scratch, predict their effectiveness and safety before they are even synthesized, and streamline the complex logistics of clinical trials, making them faster and more efficient. By automating and accelerating these critical steps, LLMs have the potential to dramatically reduce the time and cost associated with bringing new drugs to patients, ultimately transforming how we discover and develop future therapies.

## Teaser

In this review, the authors explore the transformative impact of large language models (LLMs) on drug discovery and development. They detail how LLMs can potentially accelerate our understanding of disease mechanisms, facilitate *de novo* drug discovery, and optimize clinical trial processes, including patient matching and outcome prediction. They also assess the current maturity of LLM applications and outline future directions for integrating these advanced computational models into the pharmaceutical pipeline.

## Introduction

“Language is only the instrument of science, and words are but the signs of ideas.”—Samuel Johnson (*A Dictionary of the English Language*, preface)

The pursuit of new drugs to research and develop is a long-term commitment that typically takes 10–15 years and costs over $2 billion in order to bring a new drug to a patient.^1^ This procedure is traditionally divided into three stages: understanding the disease and selecting a treatment target, developing targeted therapies, and testing their effectiveness in clinical trials. Each phase is time consuming and resource intensive due to the complexity of biological systems and the extensive review required. While this process is essential to minimize harm and ensure that only safe and effective therapies that have been proven to improve and extend human life are introduced, its duration can delay patient access to promising treatments. Consequently, there are extraordinary dividends to be reaped by introducing efficiency and expanding the capabilities of current practices.

Artificial intelligence (AI) tools have become essential for accelerating drug discovery and development. Among them, large language models (LLMs), also referred to as pretrained language models, are distinguished by their capacity to interpret scientific language and perform critical tasks in the drug discovery process. For example, Geneformer,^2^ which was pretrained on 30 million single-cell transcriptomes, aids in disease modeling and has successfully identified therapeutic targets for cardiomyopathy through *in silico* deletion. Researchers have demonstrated that LLMs such as Chemcrow^3^ and Coscientist^4^ have the potential to automate chemistry experiments, particularly in directed synthesis and chemical reaction prediction. Other models, including LLM4SD,^5^ can directly analyze raw experimental data to perform scientific synthesis, inference, and hypothesis generation, aligning with human expert analysis. Med-PaLM,^6^ a large-scale LLM incorporating clinical knowledge, has even surpassed human experts on United States Medical Licensing Examination (USMLE)-style medical questions, illustrating the potential of LLMs to reduce the burden of clinical-trial tasks.

**Figure 1:**
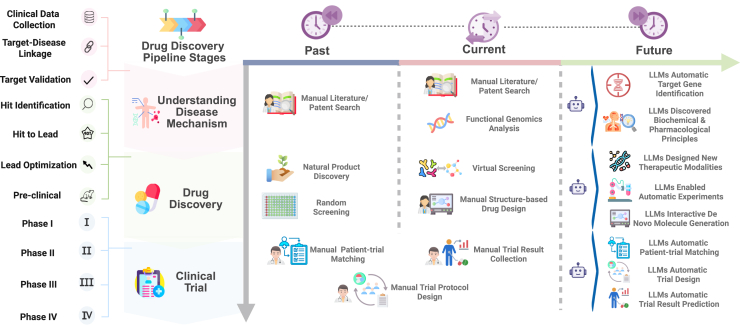
Large language models shaping the future landscape of drug discovery and development In the past, drug discovery relied on labor-intensive manual processes, demanding significant human effort and resources. Nowadays, advances in biotechnology, AI, and *in silico* tools have reduced these burdens, though full automation remains limited, especially in clinical trials where design and participant matching still depend on human expertise. In the future, the continued development of large language models (LLMs) is expected to drive a highly automated drug discovery pipeline, accelerating breakthroughs and efficiency.

Advances in LLMs have the potential to transform the drug discovery pipeline, enabling highly automated applications across all three stages (Figure 1). In the initial stage, LLMs may assist in clarifying disease mechanisms and identifying potential targets.^2^^,^^7^^,^^8^ By performing functional genomics analysis, they can pinpoint genes with desirable characteristics for drug targeting, drawing on experimental data and gene-related literature. LLMs may also uncover new insights into biochemistry and pharmacology through literature review. In the drug discovery phase, LLMs may automate chemistry experiments, control robotic systems, and suggest novel molecules through interactive platforms for compound generation and editing.^4^ During clinical trials, LLMs may streamline patient matching^9^ and trial design^10^ by analyzing profiles and requirements, while early research suggests they may predict trial outcomes^11^^,^^12^ by analyzing historical data.

In this paper, we first provide background information on LLMs and explore two paradigms of applying LLMs to drug discovery and development (see Box 1): *specialized models* trained on scientific language and *general-purpose models* trained on broader textual data. We further categorize existing approaches into three methodological classes (see Box 1): language model (LM)-based (≤100 million parameters), LLM-based (>100 million parameters), and hybrid-LM/LLM models.

Box 1 **Background, paradigms, and methods of LLMs**

**Background of LLMs**

LLMs are Transformer-based neural networks that are pretrained, e.g., next-token prediction^13^ or masked-token reconstruction,^14^ on massive corpora (from general text to domain-specific formats like SMILES or FASTA). This training enables LLMs to model the relationships between words and concepts based on context, allowing them to understand and generate coherent text. Once pretrained (often with more than 100 million parameters), these models can be fine-tuned for a broad array of downstream tasks, from question answering^15^ to specialized scientific workflows. Due to their scale and complexity, LLMs typically rely on GPU acceleration for both training and inference.

Today’s LLM landscape is a mix of both commercial and open-source efforts. Major tech companies, e.g., OpenAI and Google, produce proprietary, API-driven models (e.g., ChatGPT^13^ and Gemini) under closed licenses. In parallel, vibrant open-source communities, e.g., Meta and DeepSeek, provide freely available models (e.g., Galactica,^16^ Llama,^17^ DeepSeek^18^), encouraging transparency and on-premises deployment.

**Main Paradigms**

**Figure 2:**
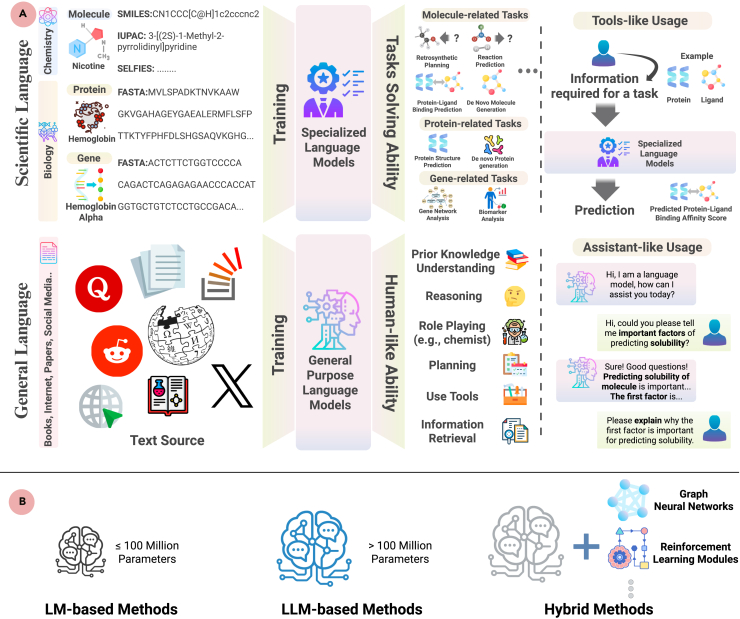
Main paradigms and types of LLMs in drug discovery and development (A) The two main paradigms of language models. Specialized models decode scientific languages for targeted tasks, while general-purpose models act as conversational assistants trained on diverse text sources. (B) Three types of LLM methods include LM-based, LLM-based, and hybrid-based methods.

Two learning paradigms of LLMs are illustrated in Figure 2A.

(1) Specialized language models: These models are trained on domain-specific scientific language, e.g., SMILES^19^ for small molecules and FASTA for proteins and polynucleotides. They learn statistical patterns from raw biochemical and genomic data and can perform tasks involving molecules, proteins, and genes. For example, when provided with a ligand’s SMILES string and a protein’s amino acid sequence, these models can predict protein-ligand binding affinities.^20^
(2) General-purpose language models: These models are pretrained on vast and diverse text collections, including scientific literature, web pages, and books.^13^ This extensive training equips them with capabilities such as reasoning, planning, tool use, information retrieval, and role playing in scientific scenarios.^3^^,^^4^^,^^5^ In practice, users interact with these models as conversational assistants to address and solve specific problems.

**Methods**

Different LLM methods for drug discovery and development are illustrated in Figure 2B.

(1) LM-based methods: These leverage smaller language models (LMs), usually of less than 100 million parameters, which are normally trained on domain-specific corpora, e.g., SMILES,^21^ FASTA,^22^ and biomedical literature,^23^^,^^24^ for downstream tasks, e.g., ADMET prediction, to extract statistical patterns. Due to limitations of size, they lack the few-/zero-shot learning and reasoning abilities of larger models.
(2) LLM-based methods: These leverage large (more than 100 million parameters) LMs, e.g., ChatGPT,^13^ ESM2,^25^ and Galactica,^16^ pretrained on vast corpora ranging from general text to domain-specific sequences like SMILES^26^ and FASTA.^25^ They can be fine-tuned for specialized tasks (e.g., ADMET prediction^26^) and may exhibit emergent capabilities, supporting few-/zero-shot applications such as protein variant effect scoring,^25^ and protein-ligand binding site identification.^20^
(3) Hybrid LM/LLM methods: These architectures leverage the complementary strengths of large language models and dedicated computational modules—such as graph neural networks for geometric reasoning, reinforcement-learning that iteratively refines solutions, and machine-learning modules. For example, generative models such as REINVENT4^27^ employ reinforcement learning to steer LM outputs toward desired biochemical properties, and LLM4SD^5^ leverages features generated by LLMs to train its machine-learning classifiers/regressors for molecular property prediction.^5^

**Figure 3:**
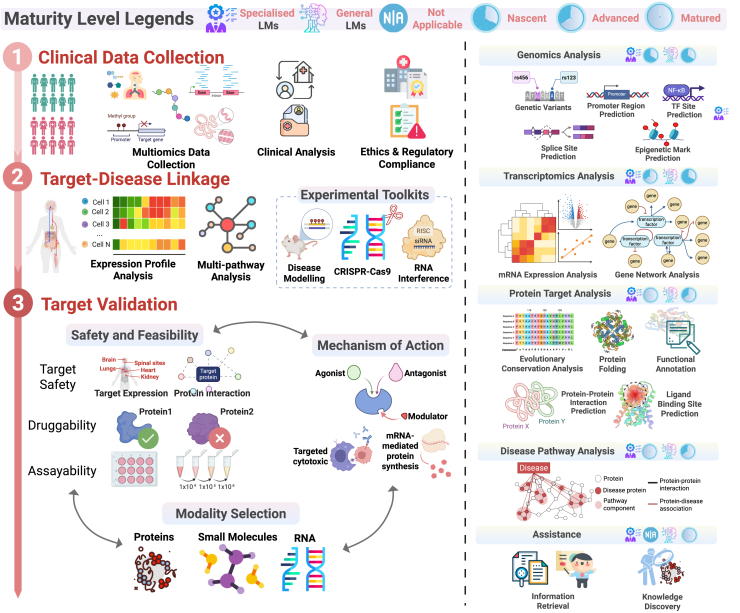
Understanding disease mechanisms The left part of the figure depicts the process of understanding disease mechanisms, which involves clinical data collection, target-disease linkage analysis, and target validation. Clinical data collection gathers patient data and identifies patient subgroups using multi-omics data, while target-disease linkage analysis explores the relationship between targets and diseases. Target validation includes safety, feasibility, mechanisms of action, and modality selection. The right part shows how LLMs assist in these tasks, including genomics and RNA analysis, pathway analysis, target profiling, and strategic profiling. Maturity levels for each task category are displayed immediately beside its title.

**Figure 4:**
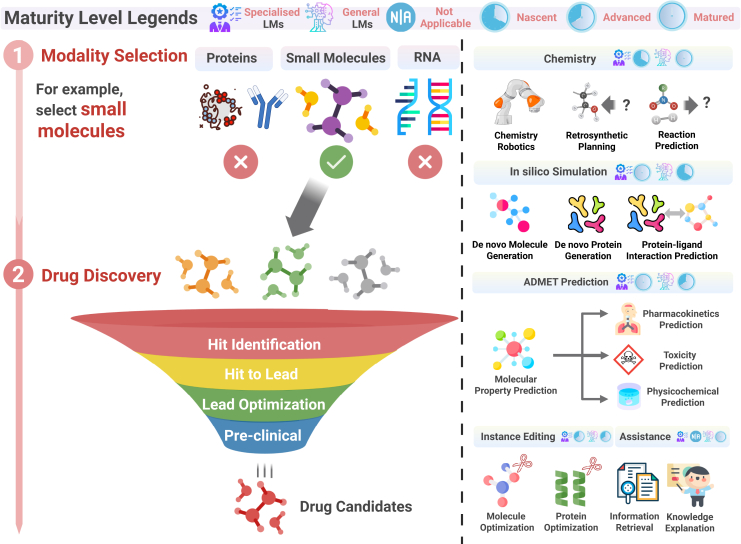
Drug discovery The left part of the figure illustrates the processes involved in drug discovery. The right part of the figure highlights the tasks that LLMs can perform to facilitate these processes. Maturity levels for each task category are displayed immediately beside its title.

We then discuss how these paradigms may be applied in the canonical stages of the drug pipeline—*understanding disease mechanisms*, *drug discovery*, and *clinical trials*—each illustrated in Figures 2, 3, and 4. On the left side of each figure, we outline the core processes within each stage, while on the right, we highlight the downstream applications to which LLMs can potentially contribute (e.g., hypothesis generation, virtual screening, patient cohort stratification).

These figures illustrate drug discovery and development pipeline stages, understanding disease mechanisms (Figure 2), drug discovery (Figure 3), and clinical trials (Figure 4), as well as a maturity assessment of two representative LLM paradigms (i.e., specialized LLMs and general LLMs) across downstream tasks in the three stages to gauge their current capabilities and limitations. The maturity levels for each downstream task are displayed on the right part of each figure, immediately adjacent to the title of each downstream task category. Specifically, we employ a *four-level maturity model* for *a clear, systematic evaluation*.

(1) Not applicable: the LLM paradigm is irrelevant or unsuitable for this task.
(2) Nascent: investigated only *in silico*; lacks real-world experimental validation.
(3) Advanced: demonstrated efficacy in laboratory or pilot studies under realistic conditions.
(4) Matured: deployed in operational environments (e.g., hospital systems or pharmaceutical pipelines) with documented impact and utility.

Finally, we conclude with a discussion of future directions, addressing ethical concerns (privacy, fairness, bias) and technical challenges (hallucinations, interpretability) critical for making LLMs trusted, efficient tools in drug discovery and patient care.

**Figure 5:**
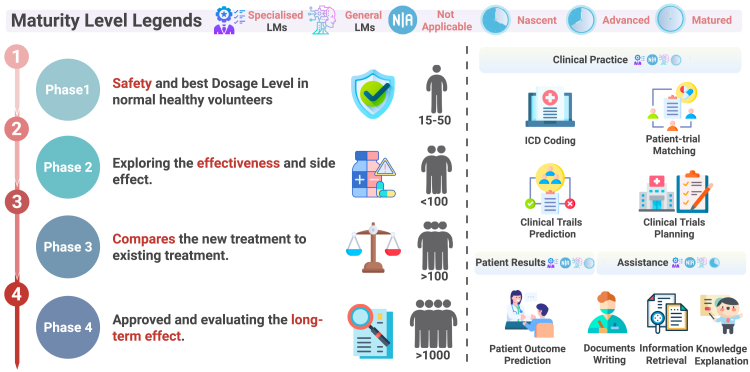
Clinical trials The left part of the figure illustrates the processes involved in clinical trials. Clinical trials consist of four phases: phase 1, phase 2, phase 3, and phase 4. The right part of the figure highlights the tasks that LLMs can perform to facilitate these processes. Maturity levels for each task category are displayed immediately beside its title.
(2) **How advanced are LLMs in facilitating downstream tasks across various *de novo* drug discovery and development stages?** To assess the maturity of LLM applications across these stages, we evaluate current applications of LLMs and classify each one into one of four categories: not applicable, nascent, advanced, and mature. These indicators provide an overview of the current state in the field and indicate promising future directions.
(3) **What are the future directions for LLMs in *de novo* drug discovery and development?** We examine the evolving role of LLMs in expanding biological use cases while addressing ethical concerns, including privacy, fairness, and bias, as LLMs are increasingly applied to sensitive health data and medical decision-making. We also discuss the technical challenges, such as hallucinations and the need for improved model interpretability. Addressing these issues will be crucial for making LLMs trusted, efficient tools in drug discovery and patient care, as explored in the section on future directions.

Overall, we aim to address three key questions for researchers and practitioners looking to leverage LLMs to enhance the drug discovery and development pipeline:

(1) **How can LLMs be effectively integrated into the various stages of *de novo* drug discovery and development?** We begin by defining the types of LLMs considered in this study (Figure 2). The drug discovery and development pipeline is categorized into three stages: *understanding disease mechanisms* (Figure 3), *drug discovery* (Figure 4), and *clinical trials* (Figure 5). Each figure outlines the processes in the left column and highlights the tasks LLMs can perform in the right column, illustrating how LLMs can optimize each stage of the pipeline.

## Understanding disease mechanisms

Understanding disease mechanisms is a critical first step in drug discovery, with the primary goal of identifying a suitable protein target for potential drugs. This process involves three main stages (Figure 3): clinical data collection, target-disease linkage analysis, and target validation. The overview of LLM tools for the *understanding disease mechanisms* step is summarized in Table 1. In the first stage, clinical data collection involves gathering patient data and categorizing individuals into subgroups, enabling the integration of clinical and multi-omics data to improve understanding of disease variations and potential differences in disease mechanisms across patient groups.^28^ The target-disease linkage phase establishes connections between potential protein targets and specific diseases through pathway analysis,^29^ gene expression profiling, and experimental techniques like CRISPR-Cas9^30^ and *in vivo* disease modeling.

**Table 1:** Overview of the “understanding disease mechanism” stage

Model/framework	Type	Method	Sub-task	Subsub-task	Input (training data type)	Output
HyenaDNA^36^	Spe	LM-based	genomic analysis	genetic variant analysis	nucleotide sequence	promoter/enhancer/TF binding site/splice site prediction; variant effect prediction; epigenomic profiling
Genslms^39^	Spe	LLM-based	genomic analysis	genetic variant analysis	nucleotide sequence	variant effect prediction
RNA-FM^41^	Spe	LLM-based	genomic analysis	genetic variant analysis	nucleotide sequence	RNA structures
Evo^40^	Spe	LLM-based	genomic analysis	genetic variant analysis; genomic regions-of-interest predictions	nucleotide sequence	variant effect prediction
Dnabert-2^38^	Spe	LM-based	genomic analysis	genetic variant analysis; genomic regions-of-interest predictions	nucleotide sequence	promoter/enhancer/TF binding site prediction; splice site prediction; variant effect prediction; epigenomic profiling
RNAErnie^43^	Spe	LM-based	genomic analysis	genetic variant analysis; genomic regions-of-interest predictions	nucleotide sequence	RNA-RNA interaction prediction; RNA secondary structure prediction
RiNALMo^42^	Spe	LM-based	genomic analysis	genetic variant analysis; genomic regions-of-interest predictions	nucleotide sequence	RNA structures; splice site prediction
Geneformer^2^	Spe	LM-based	transcriptomics analysis	mRNA expression analysis; gene network analysis	gene sequence	cell type annotation; cell perturbation prediction
scGPT^8^	Spe	LLM-based	transcriptomics analysis	mRNA expression analysis; gene network analysis	gene sequence	cell type annotation
scMulan^7^	Spe	LLM-based	transcriptomics analysis	mRNA expression analysis; gene network analysis	gene sequence	cell type annotation
scFoundation^46^	Spe	LLM-based	transcriptomics analysis	mRNA expression analysis; gene network analysis	gene sequence	cell type annotation; cell perturbation prediction; drug response prediction
scBERT^49^	Spe	LM-based	transcriptomics analysis	mRNA expression analysis; gene network analysis	gene sequence	cell type annotation
cellPLM^50^	Spe	LM-based	transcriptomics analysis	mRNA expression analysis; gene network analysis	gene sequence	cell type annotation; spatial transcriptomic imputation
GeneCompass^48^	Spe	LLM-based	transcriptomics analysis	mRNA expression analysis; gene network analysis	gene sequence	cell type annotation; spatial transcriptomic imputation
ProstT5^59^	Spe	LLM-based	protein target analysis	evolutionary conservation	protein sequence	protein embeddings; protein contact maps; protein function
Ankh^153^	Spe	LLM-based	protein target analysis	evolutionary conservation	protein sequence	protein embeddings; protein contact maps; protein function
xTrimoPGLM^57^	Spe	LLM-based	protein target analysis	evolutionary conservation	protein sequence	protein embeddings; protein contact maps; protein function
gLM2^58^	Spe	LLM-based	protein target analysis	evolutionary conservation	nucleotide and protein sequence	protein embeddings; protein contact maps; protein function
ESM^52^	Spe	LLM-based	protein target analysis	evolutionary conservation; protein folding; functional annotation	protein sequences	protein embeddings; protein function
ESM-1v^54^	Spe	LLM-based	protein target analysis	evolutionary conservation	protein sequence	protein embeddings; protein contact maps; protein function; variant effect prediction
AlphaFold2^61^	Spe	hybrid-LM	protein target analysis	protein folding	protein sequence	protein structure
AlphaFold3^65^	Spe	hybrid-LLM	protein target analysis	protein folding	protein/gene sequences, SMILES	3D biomolecular complex structure
Openfold^62^	Spe	hybrid-LM	protein target analysis	protein folding	protein sequence	protein structure
RGN^66^	Spe	hybrid-LLM	protein target analysis	protein folding	protein sequence	protein structure
RosettaFold-AA^64^	Spe	hybrid-LLM	protein target analysis	protein folding; protein-ligand interaction and binding site prediction	biomolecular sequence	biomolecular (complex) structures
ESM2/ESMFold^25^	Spe	LLM/hybrid-LLM	protein target analysis	functional annotation	protein sequence	protein structure
TAPE^67^	Spe	LM-based	protein target analysis	functional annotation	protein sequence	protein embeddings; protein contact maps; protein function
Precogx^69^	Spe	hybrid-LLM	protein target analysis	functional annotation	protein sequence	protein embeddings; protein contact maps; protein function
ProteinChat^70^	Spe	LLM-based	protein target analysis	functional annotation	natural text, protein sequence, protein structure	protein function
ESM3^71^	Spe	hybrid-LLM	protein target analysis	functional annotation	protein sequence, structure, function	protein sequence, structure, function
Deep-prosite^72^	Spe	hybrid-LLM	protein target analysis	protein-ligand interaction and binding site prediction	protein sequence	binding site residues
DockGPT^73^	Spe	hybrid-LLM	protein target analysis	protein-ligand interaction and binding site prediction	protein sequence and structure	protein-protein complex

“Spe” and “Gen” refer to specialized and general, respectively. ESM2 is LLM based, whereas ESMFold is hybrid-LLM based.

Target validation is an ongoing, iterative process that does not have a fixed starting point.^31^ This cycle includes assessing the mechanism of action, selecting the most appropriate therapeutic modality, and evaluating safety and feasibility. The safety and feasibility assessment involves examining the potential organismal impact, druggability of the target, and the practicality of assays for feasibility.^32^ This flexible approach ensures that targets are continuously evaluated for viability and safety before advancing further in the drug development pipeline.

### Genomics analysis

As shown in Figure 3, genomic analysis underpins the early phases of disease mechanism investigation, specifically the *clinical data collection* and *target-disease linkage* phases. Decades of genome-wide association studies (GWASs) have identified key genomic regions linked to various diseases, advancing genomic analysis for disease understanding and target discovery.^33^ Integrating genetic associations into drug discovery has the potential to increase the success rate of clinical targets.^34^ In the clinical data collection phase, genomic data are used to classify patient subgroups by identifying shared variants and molecular traits. In the target-disease linkage phase, specific variants and regulatory elements provide mechanistic insight into how these subtypes may drive disease.

Recent advances in nucleotide-specific LLMs, such as DNA-BERT,^22^ Nucleotide Transformer,^35^ and HyenaDNA,^36^ have enabled scalable interpretation of genomic information by leveraging structural parallels between genomic sequences and human language. Building on the idea that DNA follows language-like patterns, *genetic variant analysis* uses LLMs to identify functional alterations—such as SNPs or insertions or deletions (indels)—that may drive disease phenotypes. By applying masked language modeling to nucleotide sequences, these LLMs^22^^,^^35^ learn to recognize patterns associated with functionally significant variants, including SNPs, insertions, and deletions.^37^ DNA-BERT^22^ and Nucleotide Transformer^35^ can detect conserved motifs and prioritize variants that may contribute to disease, informing the identification of subtype-specific drivers. Nucleotide Transformer has been applied in the classification of SARS-CoV-2 variants^38^ and the study of viral evolution.^39^ The recently developed HyenaDNA model^36^ enables long-range variant modeling across sequences of up to 1 million tokens, overcoming quadratic attention limitations in earlier models. Similarly, Evo^40^ incorporates deep signal processing to predict variant functionality at single-nucleotide resolution across whole genomes. In parallel with DNA modeling, RNA-focused LLMs have enhanced the functional analysis of RNA structure and expression regulation, relevant to both subtyping and mechanistic modeling. RNA-FM,^41^ trained on over 23.7 million RNA sequences, enables precise secondary structure prediction. RiNALMo^42^ and RNAErnie^43^ improve generalization across unseen RNA families. Collectively, these models form the foundation for understanding how sequence-level variation underlies disease phenotypes.

A further application of nucleotide LLMs is in predicting *genomic regions of regulatory interest*, such as promoter regions, transcription factor (TF) binding sites, and splice sites—key elements involved in gene regulation and often disrupted in disease. Specialized nucleotide LLMs, fine-tuned on large datasets and domain-specific knowledge, have shown improved performance in predicting such regions compared to previous methods.^35^^,^^38^ These capabilities support both disease modeling and candidate target evaluation during the target-disease linkage stage. Similarly, epigenetic marks, including DNA methylation and histone modifications, regulate gene expression without altering the DNA sequence and play a key role in disease and therapy. Accurately predicting these marks is essential for understanding their impact on gene expression and disease. The Evo model,^40^ leveraging deep signal processing, achieves single-nucleotide resolution over long genomic sequences, improving regulatory element prediction. Additionally, RNA-specific LLMs, such as RNAErnie^43^ and RiNALMo,^42^ enhance RNA regulatory region and splice site prediction, demonstrating strong generalization to unseen RNA families. Nucleotide LLMs have also been fine-tuned to predict individual histone modifications, such as H3K14ac, H3K36me3, and H3K4me1, in an effort to address the challenges posed by the complexity and variability of epigenetic marks.^38^

Although current nucleotide LLMs detect functional variants and regulatory elements, future models should integrate multi-omic and spatial context to achieve tissue specificity, enable causal inference and the generative design of regulatory sequences, provide interpretable motif insights, and support real-time clinical variant annotation.

#### Maturity assessment

Specialized LLMs have recently been developed to encode nucleotide sequences^22^^,^^35^ for applications such as genetic variant analysis.^38^ However, they remain in the early stages, requiring further validation. Similarly, general LLMs, still nascent, show potential but need improvement in tasks like explaining evolutionary processes or designing DNA sequences,^44^ highlighting the ongoing evolution and future promise of the field.

### Transcriptomics analysis

In the disease mechanism framework (Figure 3), transcriptomic data is essential for both *clinical data collection* and *target-disease linkage*, as it captures cell-type-specific gene expression changes that define disease states. Transcriptomics—focused on quantifying RNA expression across tissues—has benefited from advances in single-cell and high-throughput sequencing technologies, yielding datasets that detail cellular behaviors at unprecedented resolution. However, data for rare diseases or inaccessible tissues are often sparse, limiting robust model development.^45^ To address this, specialized gene LLMs, such as Geneformer,^2^ use techniques like “rank value encoding” to map single-cell transcriptomes into ranked gene sequences, normalizing expression levels across tissues. This enables consistent comparison of gene expression patterns across conditions, supporting patient stratification and functional profiling. Models like Geneformer,^2^ scGPT,^8^ scMulan,^7^ and scFoundation^46^ excel in analyzing sparse data, modeling gene networks, and understanding complex interactions beyond simple cell-level annotations. In addition, literature-trained models such as PWAS^47^ offer a complementary route to *target-disease linkage* by associating genes and diseases based on publication data, capturing early therapeutic signals even before experimental validation.

A key challenge in transcriptomic analysis is accurate *mRNA expression analysis* in low-sample or noisy contexts. In contrast, specialized models like Geneformer^2^ leverage pretraining on transcriptomic data to efficiently adapt to specific disease contexts. Geneformer demonstrates superior performance in gene network analysis with minimal data, as evidenced by its ability to distinguish key factors in the NOTCH1-dependent network from just 884 endothelial cells from healthy versus dilated aortas, outperforming methods that relied on a dataset of approximately 30,000 cells.^2^ Similarly, scGPT,^8^ a pre-trained transformer model for single-cell multi-omics analysis, can generate meaningful cell-type clusters in a zero-shot manner, without additional fine-tuning. These capabilities are critical for clinical subtyping in the *clinical data collection* step, where high-resolution cell-type differences can delineate patient groups. Complementing these approaches, GeneCompass^48^ enhances cross-species transcriptomic analysis by integrating regulatory networks and prior biological knowledge, significantly improving gene expression predictions and functional annotation. Moreover, scBERT^49^ and CellPLM^50^ further refine single-cell RNA analysis by capturing intricate gene-gene and cell-cell interactions, addressing challenges related to batch effects and cell state characterization.

*Gene network analysis* is essential for uncovering disease mechanisms and informing *target validation* by characterizing regulatory relationships between genes. However, uncovering regulatory elements that modulate these networks is challenging, particularly for rare diseases or conditions involving clinically inaccessible tissues.^2^^,^^8^ To address this challenge, Geneformer^2^ applies a deep learning approach that identifies directional relationships between genes by learning which genes influence or are influenced by others through observed expression patterns. This process automatically constructs gene networks by mapping gene interactions. Geneformer also employs *in silico* deletion, a computational technique that simulates the removal of individual genes to evaluate their functional importance within the network. In contrast, scGPT^8^ constructs gene networks through computational embeddings that capture gene similarity based on co-expression and functional patterns. These network representations facilitate the identification of key regulatory nodes and potential therapeutic targets. Expanding on these capabilities, GeneCompass^48^ integrates cross-species transcriptomic data, enabling the identification of conserved patterns in gene regulation, while CellPLM^50^ explicitly models cell-cell interactions to improve network inference. Additionally, scBERT^49^ refines gene network analysis by leveraging large-scale single-cell RNA-seq data to learn more generalizable and interpretable representations of gene interactions.

Though current transcriptomic LLMs can identify cell types, infer gene networks, and predict expression from sparse data, future models should integrate spatial and proteomic context to map cell-cell interactions, capture temporal trajectories and lineage dynamics, enable causal inference and *in silico* perturbation design, provide interpretable gene-level attributions to guide hypothesis testing, and generalize to rare or cross-species cell states for broader applicability.

#### Maturity assessment

Specialized LLMs, such as Geneformer,^2^ have made significant strides in gene network analysis, successfully distinguishing between normal and cardiomyopathic cardiomyocytes. This enabled the identification of key genes linked to hypertrophic and dilated cardiomyopathy, with targets like ADCY5 and SRPK3 validated through experimental induced pluripotent stem cell (iPSC)-derived cardiac microtissues with Titin truncating mutations.^2^ Thus, specialized LLMs have advanced in transcriptomic data analysis and disease mechanism deciphering. In contrast, general LLMs are still in the nascent stage for transcriptomic analysis, with ongoing research exploring tasks like automating cell type analysis^51^ and data analysis via code generation.^44^

### Protein target analysis

In the final stage of disease mechanism modeling (Figure 3), protein-level analysis plays a critical role in *target validation*, assessing the structure, function, and druggability of candidate proteins. Protein sequences are often the most readily available information about a candidate target and serve as the foundation for functional inference and therapeutic assessment. Specialized LLMs are particularly valuable in this context, as they can provide extensive analyses, including evolutionary conservation, functional annotation, protein folding, and binding-site prediction. These models can extract relevant information from sequence data alone, enabling the characterization of biological traits and functions even in the absence of experimental data, such as 3D protein structures.

One foundational application is *evolutionary conservation*. Models such as ESM^52^ exploit the statistical regularities in protein sequences shaped by evolution, allowing them to infer biologically meaningful constraints. Mutations that enhance an organism’s fitness are more likely to be selected by evolutionary forces, leading to unique sequence signatures.^53^ These sequence patterns reflect conserved functional sites, and LLMs trained with masked amino acids can predict mutation tolerance, revealing which residues are essential for structure or function. Research has shown the efficacy of this approach, as demonstrated by the ESM language model’s ability to accurately predict mutational effects across various proteins without additional training.^54^ Earlier work, such as UniRep,^55^ demonstrated how deep sequence-based representation learning enables efficient exploration of evolutionary fitness landscapes, improving stability and functional annotation predictions. Expanding on this, Ankh^56^ introduces an optimized transformer model that balances model size and efficiency, outperforming existing models in evolutionary conservation analysis. Additionally, xTrimoPGLM^57^ extends large-scale protein modeling by integrating bidirectional autoencoding and autoregressive training to improve both structure prediction and mutation effect analysis. GLM2,^58^ leveraging metagenomic diversity, enhances functional representation learning, aiding in evolutionary insights at the protein level. Moreover, ProstT5^59^ advances conservation analysis by integrating protein sequence and structure into a unified representation, refining predictions of functional residues and evolutionary constraints. These conservation-focused models are critical for assessing whether a candidate target is robust to mutation and functionally central, which are key criteria for validating its relevance in therapeutic development.

Closely linked to conservation analysis is *protein folding*, a fundamental task in structure-based target validation. Since function often depends on three-dimensional conformation, structure prediction from sequence is essential for understanding target accessibility, binding interfaces, and interaction specificity. LLMs trained on protein sequence data can capture these evolutionary trends, as demonstrated by ESM,^52^ which accurately decodes protein structure from sequence data. By generating pairwise interaction maps (attention matrices) between amino acid positions, these models can predict amino acid contacts with remarkable precision, suggesting that structural information can be inferred directly from sequence data, consistent with Anfinsen’s dogma.^60^ Building on this foundation, AlphaFold2^61^ has revolutionized protein structure prediction, achieving atom-level accuracy even in the absence of known homologous structures. AlphaFold2’s Evoformer component, similar to the multiple sequence alignment (MSA)-Transformer approach, uses masked sequence data from MSAs to incorporate evolutionary information and predict protein structures with near-experimental accuracy.^62^ This approach has been extended to biomolecular interactions in RosettaFold,^63^ enabling the modeling of complex assemblies such as protein-protein and protein-DNA/RNA complexes.^64^ More recently, AlphaFold3^65^ has significantly enhanced biomolecular interaction modeling by incorporating small molecules, ions, and modified residues, achieving state-of-the-art accuracy in protein-ligand and protein-nucleic acid interactions. In parallel, RGN2’s ProtBERT^66^ encodes protein sequences and predicts structure without relying on MSAs, even outperforming AlphaFold2^61^ in predicting the structure of orphan proteins lacking sequence homologs.^66^ These models facilitate a comprehensive and systematic evaluation of candidate targets’ structural features, thereby informing druggability assessments and interaction modeling, which are critical elements of target validation.

To support *target validation*, another essential application is *functional annotation*, which assigns biological meaning to protein sequences by predicting their roles in cellular processes. This task is particularly important for novel or poorly characterized proteins emerging from high-throughput studies. Early LLMs trained on protein sequences^25^^,^^52^^,^^67^ demonstrated the capacity to capture meaningful sequence patterns, structural motifs, and evolutionary signals. These models generate embeddings that represent proteins in a way that preserves functional and structural similarities. This capability has been integrated into tools such as NetGO 3.0,^68^ which combine LLM-based embeddings with network information to predict protein functions across various species, thereby reducing dependence on labor-intensive experimental assays. For instance, PRECOGx^69^ used ESM^52^ to analyze G protein-coupled receptor (GPCR) sequences, revealing that interaction mechanisms of protein variants are driven by alternative splicing. These insights help identify mechanisms of dysfunction and support the prioritization of variant-bearing targets. Advances in LLMs have also led to ProteinChat,^70^ an interactive platform for exploring protein sequences, with recent studies highlighting GPT-4’s expertise in protein understanding.^44^ While protein language models were previously limited, the release of ESM2^25^ and ESM3,^71^ with 15 billion and 98 billion parameters, respectively, has significantly improved the modeling of sequence-structure-function relationships. ESMFold,^25^ based on the ESM framework, has shown remarkable precision in predicting protein structures, achieving accuracy comparable to AlphaFold2 using only a single input sequence rather than MSAs, thereby enhancing efficiency and accessibility. These models advance the field of functional annotation by enabling high-resolution, structure-aware predictions, even in the absence of experimentally determined structural data.

Ultimately, determining the druggability of a protein target—its capacity to bind small molecules or biologics—constitutes a central aspect of the *target validation* process. Protein-based LLMs have shown success in predicting protein-protein interactions,^72^ which are essential for identifying target proteins and designing biologic drugs. Notably, DockGPT,^73^ an innovative protein docking method, excels in handling conformational flexibility and binding-site information, offering high accuracy in antibody-antigen complex predictions and co-designing antibody sequences targeting specific epitopes. Additionally, RosettaFold All-Atom^64^ has enhanced protein-ligand interaction modeling by incorporating various ligands, including small molecules, metal ions, and nucleic acids, allowing for highly accurate predictions of protein-ligand complexes. These models identify binding sites and predict the effects of mutations on binding affinity, which are essential for detecting viable drug-binding pockets. Their predictive capacity streamlines drug candidate screening and design, linking sequence-based target nomination with interaction-based validation.

Though current protein LLMs can infer evolutionary constraints, predict structures, and annotate functions, future models should integrate conformational dynamics and post-translational modifications to capture protein ensembles, enable *de novo* design of enzymes, biologics, and small-molecule binders, predict allosteric and cryptic sites, incorporate high-resolution proteomics and cellular context for interaction kinetics, and extract interpretable functional motifs.

#### Maturity assessment

Specialized LLMs, such as AlphaFold2^61^ and AlphaFold3,^65^ have made significant strides in protein target analysis, with applications in structure-based drug discovery and vaccine development.^74^ AlphaFold’s success includes the rapid development of a first-in-class hit molecule for CDK20 within 30 days and the synthesis of only seven compounds.^75^ Additionally, ESM,^52^ a protein language model, has been developed to analyze GPCR proteins and identify compounds with subnanomolar affinity.^69^^,^^76^ In contrast, general LLMs like GPT-4^13^ are still in the nascent stage for protein target analysis, with models like ProteinChat^70^ showing potential in labeling protein structures but lacking extensive real-world validation. Thus, while progress has been made, the field remains in development.

### Disease pathway analysis

Pathway analysis serves as a critical step in the *target-disease linkage* process, enabling researchers to connect candidate genes or variants to broader biological functions and disease pathways. Gene regulatory network analysis is a crucial tool in pathway analysis for deciphering complex disease pathways, with general LLMs offering significant advantages. Unlike specialized models limited to sequence data, general-purpose LLMs incorporate vast scientific literature and structured databases, allowing them to reason across diverse biological contexts.^13^^,^^16^ Their interactive capabilities enable deeper engagement with complex data,^13^^,^^77^ facilitating the exploration of scientific findings. For instance, a recent study demonstrated the effectiveness of general LLMs like GPT-4 in analyzing blood transcriptional modules related to erythroid cells, where the models automatically generated gene network codes, summarized candidate genes, created reports, and fact-checked against the literature.^78^ This illustrates how LLMs can assist in pathway assembly, candidate gene prioritization, and hypothesis generation—core tasks in connecting molecular evidence to disease phenotypes. Moreover, by grounding predictions in existing knowledge, these models reduce false positives and increase the interpretability of pathway relationships. As a result, general LLMs are emerging as powerful tools that complement omics-based analyses with dynamic, literature-aware insights, enhancing the interpretability and actionability of disease pathways.

Though current LLMs aid pathway assembly and gene prioritization, future models should support dynamic, cell-type-specific pathway modeling and enable causal inference. They should also link pathway insights directly to clinical outcomes and therapies.

#### Maturity assessment

Specialized LLMs have advanced significantly in disease pathway analysis, particularly in genomics, transcriptomics, and protein target analysis. A notable breakthrough is Geneformer,^2^ a transcriptomic LLM used for gene network analysis, which has been experimentally validated and demonstrates the potential of these models in dissecting disease pathways. Similarly, general LLMs have made strides in this area, with Insilico Medicine integrating ChatGPT into its PandaOmics platform for disease pathway analysis.^79^ However, while these tools show promise, their widespread adoption is still in progress.

### Assistance

As illustrated in Figure 3, general-purpose LLMs serve as a cross-cutting assistant throughout the disease mechanism pipeline, from *clinical data collection* to *target validation*. Because the investigation of disease mechanisms spans multiple domains—from genomics to pharmacology—general-purpose LLMs, with their interactive reasoning and multimodal input handling, provide versatile support across research stages.^16^ These models excel in information retrieval, offering fast, accurate responses and tailored explanations, while also organizing large datasets to enhance workflow and productivity.^77^

At early stages, LLMs can assist in variant interpretation, generate pathway hypotheses, and recommend relevant literature. As analysis progresses, they can synthesize cross-modal findings such as transcriptomic and structural data, support interactive query refinement, and identify inconsistencies in candidate targets. By integrating with search engines, recent LLMs provide real-time access to scientific data, facilitating hypothesis generation and validation. Their ability to translate technical content into domain-adapted summaries improves cross-disciplinary communication, helping bridge gaps between computational scientists, biologists, and clinicians. As such, LLMs do not replace domain expertise but enhance it, accelerating the iteration cycle between data, interpretation, and decision-making.

#### Maturity assessment

General LLMs have reached a mature stage, significantly aiding disease mechanism research by mining and synthesizing vast scientific and medical literature.^44^^,^^78^^,^^79^ Their ability to create and interpret knowledge graphs^79^ is key in mapping gene networks and understanding gene-disease relationships.^23^ Furthermore, these models simplify complex medical and genetic concepts,^77^ enhancing both accessibility and communication in the medical field.

## Drug discovery

The drug discovery process consists of several key steps as depicted in Figure 4: hit identification, hit to lead, lead optimization, and preclinical development. The overview of LLM tools for the *drug discovery* step is summarized in Table 2. It begins with “hit identification,” where compounds with potential therapeutic effects are identified, followed by “hit to lead,” which refines the selection to the most promising candidates. In “lead optimization,” the efficacy, stability, and safety of the lead compound are enhanced. Finally, “preclinical development” involves testing the optimized compound in animal models to assess its suitability for human trials. This survey will first outline the tasks associated with each step and then explore how LLMs can be integrated to advance the drug discovery process.

**Table 2:** Overview of methods in the “drug discovery” stage

Model/Framework	Type	Method	Sub-task	Subsub-task	Input (training data type)	Output
CLARify^81^	Gen	LLM-based	chemistry	chemistry robotics	instructions and environmental data	a structured and executable plan
Inagaki LLM^84^	Gen	LLM-based	chemistry	chemistry robotics	experiment instructions	validated Python scripts for OT-II robotic execution
Coscientist^4^	Gen	LLM-based	chemistry	chemistry robotics	experiment instructions	experimental procedures and results
ChemCrow^3^	Gen	LLM-based	chemistry	chemistry robotics, retrosynthetic planning, and reaction prediction	chemical tasks and queries	plans and execution instructions for chemistry experiments
Jablonka et al.^85^	Spe	LLM-based	chemistry	retrosynthetic planning and reaction prediction	chemistry queries and datasets	predicted chemical properties, synthesis plans, and material designs
ESM^52^	Spe	LLM-based	*in silico* simulation, lead optimization	*de novo* protein generation, protein optimization	protein sequence	protein embeddings, protein contact maps, protein function
Lingo3DMol^90^	Spe	hybrid-LLM	*in silico* simulation	*de novo* molecule generation	pocket	ligand
Reinvent 4^27^	Spe	hybrid-LM	*in silico* simulation, lead optimization	*de novo* molecule generation, molecular optimization	molecular design constraints and target properties	molecular structures with optimized properties
MolGPT^87^	Spe	LM-based	*in silico* simulation	*de novo* molecule generation	SMILES strings	generated molecules with desired properties
MolT5^88^	Gen	LLM-based	*in silico* simulation, ADMET prediction	*de novo* molecule generation	SMILES strings or natural language descriptions	generated molecular descriptions or new molecular structures
GIT-Mol^89^	Gen	hybrid-LLM	*in silico* simulation, ADMET prediction	*de novo* molecule generation	molecular graphs, images, and text descriptions	molecular properties, generated molecules, and textual descriptions
Pocketgen^98^	Spe	hybrid-LM	*in silico* simulation	*de novo* protein generation	ligands, residue	protein pocket
Rita^92^	Spe	hybrid-LLM	*in silico* simulation	*de novo* protein generation	protein sequences	protein sequences with desired properties
Protgpt2^91^	Spe	LLM-based	*in silico* simulation	*de novo* protein generation	–	–
Progen2^93^	Spe	LLM-based	*in silico* simulation	*de novo* protein generation	protein sequences	*de novo* protein sequences
ProGen^96^	Spe	LLM-based	*in silico* simulation	*de novo* protein generation	protein sequences	functional protein sequences across diverse families
RFDesign^94^	Spe	hybrid-LM	*in silico* simulation, lead optimization	*de novo* protein generation, protein optimization	functional protein sites	designed protein scaffolds with embedded functional sites
RFDiffusion^95^	Spe	hybrid-LLM	*in silico* simulation	*de novo* protein generation	molecular specifications or functional motifs	*de novo* protein structures with desired functions
PoET^97^	Spe	LLM-based	*in silico* simulation	*de novo* protein generation	molecular specifications or functional motifs	*de novo* protein sequences with desired functions
ProteinDT^99^	Gen	LLM-based	*in silico* simulation; lead optimization	*de novo* protein generation; Protein optimization	text descriptions of desired protein properties	protein sequences matching the described properties
Evo^40^	Spe	LLM-based	*in silico* simulation	*de novo* Protein Generation	nucleotide sequence	*de novo* designed protein
Evo2^154^	Spe	LLM-based	*in silico* simulation	*de novo* Protein Generation	nucleotide sequence	*de novo* designed protein
ESM3^71^	Spe	LLM-based	*in silico* simulation	*de novo* protein generation	protein sequence, structure, function	protein sequence, structure, function
PSICHIC^20^	Spe	hybrid-LLM	*in silico* simulation	protein-ligand interaction prediction	protein sequence; molecule sequence	binding affinity; functional effect; binding site residue
STAMP-DPI^103^	Spe	hybrid-LM	*in silico* simulation	protein-ligand interaction prediction	drug molecular structures and protein sequences	predicted drug-protein interaction probabilities
Molformer^105^	Spe	LLM-based	ADMET prediction	–	SMILES strings	predicted molecular properties and chemical representations
LLM4SD^5^	Gen	hybrid-LLM	ADMET prediction	–	SMILES strings	predicted molecular properties
Prompt-MolOpt^108^	Spe	hybrid-LLM	lead optimization	molecular optimization	original molecule	optimized molecules
C5T5^107^	Spe	LLM-based	lead optimization	molecular optimization	IUPAC molecular names and desired property modifications	optimized molecular structures with targeted property changes
MoleculeSTM^110^	Gen	hybrid-LLM	lead optimization	molecular optimization	molecular structures and textual descriptions	retrieved or modified molecules based on text queries
ChatDrug^111^	Gen	hybrid-LLM	lead optimization	molecular optimization	drug molecular structures and text-based modification requests	drug molecules with optimized properties
ProteinMPNN^116^	Spe	hybrid-LM	lead optimization	protein optimization	protein backbone structures	protein sequences that fold into the given structures
ProtAgents^148^	Gen	hybrid-LLM	lead optimization	protein optimization	protein backbone structures	multi-agent framework for protein design

“Spe” and “Gen” are short for specialized and general-purpose LLM.

### Chemistry

As shown in Figure 4, the drug discovery pipeline begins with the design and synthesis of novel compounds, particularly small molecules, which proceed through stages such as hit identification and lead optimization. Chemistry forms the bedrock of this early-stage process, enabling the creation of candidate molecules and the execution of critical synthesis experiments. With the integration of automated laboratories, traditional medicinal chemistry has evolved to incorporate robotic systems that can conduct complex chemical reactions and high-throughput screening. After synthesis, compounds are evaluated for activity and selectivity using pharmacological assays. LLMs are increasingly central to this transformation, serving as intelligent interfaces between human researchers and automated systems. They can generate machine-readable protocols from natural language descriptions,^3^^,^^4^ helping bridge the gap between experimental intent and execution. They also support synthesis design by recommending retrosynthetic pathways and predicting reaction outcomes. This integration enhances experimental throughput and decision-making efficiency in early drug development.

One key application is *chemistry robotics*, where LLMs convert natural language commands into code for laboratory automation platforms. General LLMs such as GPT-4^13^ and CodeLlama,^80^ trained on extensive code datasets, are well-suited for generating these plans. A notable example is CLARify,^81^ which leverages GPT-3^82^ to generate task plans in a specialized chemistry description language (XDL) based on user instructions. These plans are then executed using PDDLStream solvers, achieving higher accuracy than baseline systems like SynthReader.^83^ Additionally, GPT-4 has been used to generate Python scripts for controlling the OT-II liquid handling robot,^84^ achieving 95% success within five iterations. These advances illustrate how LLMs act as intermediaries between researchers and robotic instruments, supporting the automation of synthesis procedures illustrated in the “chemistry” section of Figure 4. An emerging area of AI research involves using LLMs as autonomous agents to create and execute scientific experiments. For example, Coscientist^4^ demonstrated how LLMs could use web search engines and vector search to gather synthesis information and generate multi-instrument systems code, successfully performing complex reactions like Suzuki and Sonogashira cross-coupling.

LLMs also contribute to *retrosynthetic planning and reaction prediction*, which are essential for mapping efficient routes from commercially available precursors to target compounds. Recently, general-purpose LLMs like Chemcrow^3^ and Coscientist^4^ have advanced this field. Chemcrow^3^ integrates a broad array of tools, including SMILES conversion, patent checking, and reaction classification, employing a four-step framework—thinking, acting, providing inputs, and analyzing results—to improve LLM performance, surpassing GPT-4 in synthesis planning tasks. Additionally, a study^85^ demonstrated that a fine-tuned GPT-3 model outperformed traditional machine learning models in low-data chemistry tasks, highlighting LLMs’ potential to advance chemical research with minimal fine-tuning, even when not initially trained on chemical data. By supporting chemical planning and prediction as illustrated in the “chemistry” panel of Figure 4, LLMs are streamlining the path from molecular design to compound generation, making them indispensable tools in modern drug discovery.

Though current LLMs may assist in translating protocols into robotic code, planning retrosynthetic routes, and predicting reaction outcomes, future models should aim to integrate real-time sensor and processing data for adaptive reaction optimization and propose novel catalysts and scaffolds. They should also enable end-to-end autonomous synthesis planning that accounts for safety, cost, and sustainability.

#### Maturity assessment

While specialized LLMs in chemistry experiments remain in their early stages, general LLMs have advanced considerably. These models are now used in complex chemistry experiments, demonstrating superior performance over specialized LLMs in tasks like retrosynthetic planning and reaction prediction due to their tool use capabilities, such as reading scientific literature and assisting in molecular synthesis.^3^^,^^4^ In real-world settings, general LLMs have shown effectiveness in synthesizing molecules and controlling robotic arms.^4^^,^^81^ Despite these advances, their widespread deployment in industries, such as pharmaceuticals, remains limited, indicating the need for further research and development to fully leverage general LLMs in chemistry experiments.

### *In silico* simulation

As depicted in Figure 4, *in silico* simulation forms a core part of the computational pipeline in drug discovery, enabling the generation and evaluation of molecular structures before physical synthesis. These simulations typically involve three main tasks: *de novo molecule generation*, *de novo protein generation*, and *protein-ligand interaction prediction*.

*De novo molecule generation* refers to the *in silico* design of novel molecular structures with potential therapeutic activity, typically classified as either unconstrained or constrained based on design constraints. Unconstrained generation explores the broader chemical space of the training set, while constrained generation focuses on molecules that satisfy specific drug-like properties such as target affinity, selectivity, absorption, distribution, metabolism, and excretion (ADME) characteristics, and synthesizability.^27^ These two approaches are essential for both exploring novel chemical scaffolds and optimizing lead candidates. Early constrained approaches used pharmacophoric features,^86^ but more recent models such as MolGPT^87^ have adopted reinforcement and curriculum learning to meet multiple design constraints. General-purpose LLMs like MolT5^88^ and GPT-4^13^ usually tackle constrained molecule generation, with MolT5 using self-supervised learning to pretrain on large text and molecular datasets. However, these models generally perform less effectively than specialized models. Multimodal approaches, such as GIT-Mol,^89^ further enhance general LLM capabilities by integrating graph, image, and text data, significantly improving constrained molecule generation tasks. Additionally, Lingo3DMol^90^ introduces a pocket-based 3D molecule generation method that combines language models with geometric deep learning, enabling more precise molecular design by incorporating spatial binding constraints. Complementing these approaches, Evo,^40^ a genomic foundation model, extends sequence-based generative design across molecular and genome scales, demonstrating zero-shot function prediction and multimodal synthesis of DNA, RNA, and protein molecules, which may support molecular design and functional optimization in drug discovery.

*De novo protein generation* involves designing protein sequences from scratch, either to explore the protein space in an unconstrained manner^91^^,^^92^^,^^93^ or to achieve specific functional objectives in a constrained manner.^94^^,^^95^ Unconstrained generation, using specialized LLMs like ProtGPT2^91^ and ProGen,^96^ has demonstrated success in generating novel sequences that resemble natural proteins, while also exploring uncharted protein space. Constrained generation, aiming to create proteins with specific functions or within protein families, leverages models like ProGen^96^ and PoET,^97^ which ensure that new sequences maintain the structural integrity of the targeted family. In drug discovery, LLMs support the design of protein binders through inverse folding techniques, contributing to the early stages of the pipeline illustrated in Figure 4, particularly during hit identification and lead optimization. RFDiffusionAA^64^ generates highly specific binding pockets by leveraging structural knowledge from models like RoseTTAFold. Recent work with PocketGen^98^ has further improved pocket-based protein generation by incorporating graph transformers and sequence refinement modules, enabling the design of high-affinity binding sites with enhanced structural consistency.

General-purpose LLMs have also emerged as effective tools in protein design. ProteinDT,^99^ for instance, integrates textual descriptions into protein design and achieves high accuracy in generating *de novo* proteins guided by text. Moreover, the ESM protein language model^52^ has been employed to design both unconstrained and constrained proteins, achieving a 67% success rate in creating functional proteins. In therapeutic applications, *de novo* designed proteins have been successfully used to neutralize lethal snake venom toxins,^100^ highlighting the potential of AI-driven protein design in developing novel therapeutics. These advances correspond to the assistance and in silico simulation tasks shown on the right side of Figure 4, where LLMs enable protein optimization, molecular property prediction, and knowledge-driven design. Building on this progress, newer models such as ESM3^71^ further improve the accuracy of complex structure prediction and functional protein design. Nonetheless, a key open question remains whether LLMs trained on natural sequences can reliably generalize to the design of unnatural proteins.

*Protein-ligand interaction prediction* is central to drug discovery, as understanding how drugs (ligands) bind to protein targets informs both screening and lead optimization. *In silico* techniques such as molecular docking and predictive machine learning models have significantly accelerated early-stage development efforts. LLMs are now increasingly employed in this context, both as the backbone of specialized systems and as components within broader predictive frameworks. These applications highlight the potential of LLMs to enhance the efficiency and effectiveness of drug discovery workflows.

Protein-specific LLMs combined with molecular fingerprints have been used for high-throughput virtual screening, identifying binders with sub-nanomolar affinity.^76^ They are also embedded in broader predictive pipelines to support tasks such as docking and binding affinity estimation.^101^^,^^102^^,^^103^ PSICHIC,^20^ for example, demonstrates that learning directly from protein sequences and ligand SMILES can surpass structure-based approaches in predicting interactions. PSICHIC can also identify protein residues and ligand atoms involved in binding, highlighting how LLMs can infer key interaction features from sequence-level data alone. General-purpose models such as Galactica^16^ extend these capabilities by predicting docking scores using broader scientific context. As LLMs increasingly integrate molecular and domain-level knowledge, they offer a path toward more scalable and precise modeling of protein-ligand interactions in drug development.

Though current models are promising for *de novo* molecule generation and binding prediction, future research should aim to enable jointly optimizing molecular design with synthetic feasibility, integrating multi-scale dynamics, and capturing conformational changes and solvent effects.

#### Maturity assessment

Specialized LLMs are increasingly applied in industry, with tools being used for protein-protein complex prediction, and expanding to protein-ligand interactions and nucleic acids. In Silico Medicine has developed Chemistry42,^104^ which utilizes specialized LLMs to identify discoidin domain receptor 1 (DDR1) kinase inhibitors and generate novel molecular structures with optimized properties, validated through *in vitro* and *in vivo* studies. Similarly, IBM’s Molformer^105^ shows promise in generating molecules for SARS-CoV-2 inhibition and antimicrobial peptides.^106^ In contrast, general LLMs are still primarily confined to *in silico* environments, facing challenges in scientific understanding and quantitative analysis. For instance, GPT-4^44^ struggles with interpreting SMILES strings and lacks the precision needed for tasks like binding affinity prediction, leading to suboptimal performance in simulations.

### ADMET prediction

As shown in Figure 4, ADMET prediction—covering absorption, distribution, metabolism, excretion, and toxicity—is a critical step during the *hit to lead* and *lead optimization* stages, helping to eliminate compounds with suboptimal pharmacokinetic or toxicological profiles. By accurately forecasting these properties *in silico*, researchers can reduce costly late-stage failures and prioritize compounds with favorable biological characteristics. ADMET property prediction draws from multiple scientific domains, including physiology, physical chemistry, biophysics, and quantum mechanics. Recent developments in LLMs have significantly enhanced predictive capabilities in this area by learning patterns from large-scale molecular and textual datasets.

Specialized LLMs are typically trained on molecular representations such as SMILES strings and optimized for tasks like property classification and toxicity assessment. Transformer-based architectures such as Molformer^105^ have established new performance benchmarks in molecular property prediction, although their training requires considerable computational resources. In parallel, general-purpose LLMs have also shown promise when adapted or augmented for ADMET-related tasks. Models like LLM4SD^5^ and Galactica^16^ can enhance traditional machine learning workflows or be fine-tuned to directly perform ADMET predictions. Additionally, pre-trained models that combine molecular and textual inputs, such as GIT-Mol^89^ and MolT5,^88^ have demonstrated versatility across molecular property tasks. GPT-4, despite being trained on general text data, has also been used to interpret and predict molecular behaviors, although its performance depends heavily on careful prompt engineering and post-processing. The diversity of strategies, ranging from molecular-specific fine-tuning to multimodal integration, illustrates the expanding role of LLMs in predictive toxicology, pharmacokinetics, and physicochemical profiling.

Despite significant advances, key challenges remain for next-generation ADMET models. Future systems should aim to generalize robustly to novel chemical scaffolds beyond training data distributions, reducing dependence on resource-intensive fine-tuning. Enhanced interpretability frameworks are crucial to elucidate the structural and mechanistic drivers behind predictions, moving beyond black-box outputs.

#### Maturity assessment

Maturity assessment provides a cloud-based platform for real-time molecular screening and efficient prediction of molecular properties. In an advanced stage, LLM4SD^5^ utilizes general-purpose LLMs like Galactica^16^ to extract meaningful hypotheses from ADMET data. These hypotheses have outperformed traditional methods, such as random forests, and are validated by pharmacologists to ensure their relevance and effectiveness.

### Lead optimization

Lead optimization involves modifying a drug candidate’s molecular structure or protein sequence to improve its potency, safety, and stability. Traditionally, this process relies on the knowledge and experience of chemists or biologists, but it is time consuming and often requires multiple attempts to achieve the desired outcome. LLMs assist by analyzing large datasets to predict how structural changes affect molecular properties, thereby guiding decision-making and reducing the number of experimental iterations.

*Molecular optimization* modifies a compound’s structure to improve efficacy, stability, and safety, with two primary approaches: uncontrolled and controlled optimization. In uncontrolled optimization, functional groups are modified while preserving the core scaffold, using model-guided strategies to enhance desired properties. In controlled optimization, users specify molecular segments for more precise modifications. Specialized LLMs have been developed to effectively support both uncontrolled and controlled molecular optimization strategies. For uncontrolled optimization, models like Reinvent 4^27^ use reinforcement learning to modify properties while maintaining scaffold integrity. In controlled optimization, models use matched molecular pair analysis, and C5T5^107^ improves performance by training on property-specific tokens rather than. Explicit molecular pairs. Prompt-MolOpt^108^ further advances molecular optimization by leveraging prompt-based embeddings to improve multiproperty optimization, excelling even in data-scarce settings through causal generalization. Multimol^109^ has recently been introduced for multi-objective molecular optimization, achieving significant improvements over baseline methods. General-purpose LLMs such as MoleculeSTM^110^ and ChatDrug^111^ incorporate human feedback to refine molecular design. MoleculeSTM uses multimodal learning to generate structures from textual descriptions, while ChatDrug refines workflows through iterative feedback. Although GPT-4^13^ demonstrates some capacity for structure innovation, its lack of iterative refinement limits its utility in fully automated pipelines.

*Protein optimization*, similar to molecular optimization, involves modifying protein structures to enhance functionality and safety, often requiring labor-intensive iterative adjustments by biochemists. LLMs contribute to this process by predicting the effects of structural modifications on protein properties, thereby streamlining early design decisions. In antibody drug development, for example, LLMs help improve antigen binding, reduce immunogenicity, enhance stability, and prevent issues such as polyspecificity.^112^^,^^113^^,^^114^ Optimization strategies can be broadly categorized as uncontrolled or controlled, each benefiting from LLM support. In uncontrolled optimization, models like ESM^52^ suggest evolutionarily viable mutations to enhance protein fitness across families,^115^ as demonstrated in immunoglobulin G (IgG) antibody affinity improvements with minimal testing. In controlled optimization, techniques such as protein hallucination and inpainting^94^ refine sequences while preserving backbone structure.^116^ Additionally, ProteinDT,^99^ a general-purpose LLM, optimizes protein sequences using prompts containing specific property information, employing latent interpolation to align text and protein representations. These advances show that LLMs can effectively optimize protein structure, stability, and binding, offering a novel approach to feature-specific protein engineering.

Though existing methods show promise, future optimization models should better navigate complex multi-objective trade-offs (e.g., potency vs. safety) and reduce reliance on extensive training data. They should integrate a deeper biological context for physiologically relevant predictions, and anticipate downstream effects beyond immediate targets.

#### Maturity assessment

Specialized LLMs for lead optimization have been validated through real-world experiments. For instance, Moret et al. (2023)^117^ developed chemical language models that identified a new phosphatidylinositol 3-kinase (PI3K) γ ligand with sub-micromolar activity, while Hie et al. (2023)^115^ enhanced the affinity of seven antibodies against Ebola and SARS-CoV-2 through a language-model-guided process. In contrast, general LLMs are still in the early stages, with *in silico* testing only. The main challenge for general LLMs in lead optimization is the need for a deep understanding of scientific language.

### Assistance

In the early stages of drug discovery, including the “understanding disease mechanisms” phase, researchers rely on diverse resources such as compound libraries, scientific publications, and patent data to inform target selection and therapeutic strategy. To access and integrate this information, general-purpose LLMs are increasingly employed in information retrieval workflows, combining web search and knowledge extraction to gather relevant data from literature, compound databases, and intellectual property sources. Models such as Galactica^16^ and GPT-4^13^ further support researchers by clarifying complex scientific concepts and enhancing domain-specific understanding, thereby improving early-stage decision-making.

#### Maturity assessment

General LLMs have reached an advanced stage in information retrieval and explanation for drug discovery. Some biotech companies are exploring ChatGPT plug-ins for searching medical answers in company documents,^79^ while GPT-4 enhances drug discovery through its coding capabilities, assisting in tasks like data downloading and preprocessing.^44^

## Clinical trials

As the final stage of drug development, clinical trials evaluate a candidate compound across four sequential phases to assess its safety, efficacy, and long-term effects, as illustrated in Figure 5. The overview of LLM tools for the *clinical trials* step is summarized in Table 3. Phase 1 tests the compound’s safety and tolerability in a small group of healthy volunteers. Phase 2 evaluates its efficacy and side effects in a larger patient group. Phase 3 compares the new treatment with existing ones in a larger patient population to identify differences. Phase 4, conducted after regulatory approval, monitors the compound’s performance under real-world conditions to detect long-term or rare adverse effects. As shown on the right side of Figure 5, LLMs can support this pipeline through various tasks, including clinical-trial prediction and planning, patient-trial matching, and outcome reporting, thereby enhancing decision-making throughout the clinical validation process.

**Table 3:** Overview of methods in the “clinical trials” stage

Model/Framework	Type	Method	Sub-task	Subsub-task	Input (training data)	Output
PLM-ICD^121^	Gen	LLM-based	clinical practice	ICD coding	clinical text from electronic health records (EHRs)	predicted ICD diagnostic codes
Med-monoT5^122^	Gen	LLM-based	clinical practice	patient-trial matching; clinical trial planning and prediction	patient descriptions from EHRs	ranked clinical trials matching patient eligibility
den Hamer et al.^123^	Gen	LLM-based	clinical practice	patient-trial matching	patient medical profiles and clinical trial eligibility criteria	pre-screening results for clinical trial matching
TrialGPT^9^	Gen	LLM-based	clinical practice	patient-trial matching	patient medical records and clinical trial eligibility criteria	ranked clinical trial matches for patient recruitment
Trial2Vec^155^	Gen	LM-based	clinical practice	clinical trial planning and prediction	clinical trial documents	trial embeddings for similarity search and outcome prediction
cliniDigest^125^	Gen	LLM-based	clinical practice	clinical trial planning and prediction	clinical trial descriptions	summarized clinical trial information
FRAMM^126^	Spe	LM-based	clinical practice	clinical trial planning and prediction	clinical trial site data with missing modalities	ranked trial site selections optimizing diversity and enrollment
HINT^10^	Spe	hybrid-LM	clinical practice	clinical trial planning and prediction	multi-modal clinical trial data (drug molecules, diseases, trial eligibility criteria)	predicted clinical trial success or failure
SPOT^11^	Spe	hybrid-LM	clinical practice	clinical trial planning and prediction	clinical trial data (diseases, treatments, eligibility criteria)	predicted success probabilities of clinical trials
MediTab^12^	Spe	LLM-based	clinical practice; atient results	clinical trial planning and prediction; patient outcome prediction	medical tabular data	predictions for patient and clinical trial outcomes
Patel et al.^127^	Gen	LLM-based	clinical practice	document writing	brief patient discharge details	structured discharge summaries
Shing et al.^128^	Gen	LLM-based	clinical practice	document writing	clinical notes	discharge summaries
Enarvi et al.^129^	Gen	LM-based	clinical practice	document writing	transcripts of patient-doctor conversations	medical reports summarizing the conversations
MedViLL^131^	Spe	LLM-based	clinical practice	document writing	medical images and radiology text reports	diagnosis classifications, image-text retrieval, and radiology reports
Med-PaLM2^132^	Gen	LLM-based	clinical practice assistance	document writing	multimodal biomedical data (text, medical images, and genomic sequences)	medical insights, diagnoses, and clinical reports
NYUTron^133^	Spe	LLM-based	patient results	patient outcome prediction	unstructured clinical notes from EHRs	predictions for clinical and operational tasks (e.g., readmission risk, mortality risk, length of stay, insurance denial)
StageNet^156^	Spe	LM-based	patient results	patient outcome prediction	EHR data	predicted health risk progression and patient subtypes
Hager et al.^137^	Spe	LLM-based	patient results	patient outcome prediction	EHRs data	evaluations of clinical decision-making
MUSK^135^	Gen	LLM-based	patient results	patient outcome prediction	clinical notes, pathology images	patient outcome prediction (melanoma relapse prediction, pan-cancer prognosis prediction and immunotherapy response prediction in lung and gastro-esophageal cancers)
MMedLlama3^134^	Gen	LLM-based	patient results	patient outcome prediction	clinical notes	patient outcome prediction
BiomedGPT^136^	Gen	LLM-based	patient results	patient outcome prediction	clinical notes, pathology images	patient outcome prediction

“Spe” and “Gen” are short for specialized and general-purpose LLM.

### Clinical practice

In clinical trials, practitioners are responsible for four key tasks: International Classification of Diseases (ICD) coding, patient-trial matching, outcome prediction, and trial planning. Each of these requires substantial domain expertise and involves analyzing complex datasets such as electronic health records (EHRs), eligibility criteria (ECs), trial protocols, and clinical outcomes. General-purpose LLMs offer promising capabilities to streamline these processes by efficiently extracting, integrating, and generating information across large-scale clinical documents.

ICD coding, a foundational but labor-intensive aspect of clinical documentation, requires precise assignment of diagnostic codes to patient records. LLMs have been used to streamline this process by analyzing large volumes of EHR data and predicting the most appropriate codes, enabling practitioners to make more informed decisions. Recent approaches to automated ICD coding have evolved from traditional long short-term memory (LSTM)-based^118^^,^^119^ architectures to transformer-driven models. For example, BERT-XML,^120^ integrates BERT pretraining with multi-label attention for more accurate code prediction, while PLM-ICD^121^ adapts domain-specific models such as BioBERT,^24^ fine-tuning them for ICD coding and using segment pooling to improve efficiency and accuracy.

*Patient-trial matching* has traditionally relied on manual review of electronic health records (EHRs) and eligibility criteria (ECs) by physicians and data analysts, a process that is both time consuming and error prone. Recent studies have explored the use of general-purpose LLMs to streamline this task. For instance, Med-monoT5^122^ has been fine-tuned for medical passage ranking, and den Hamer et al. (2023)^123^ employed InstructGPT^124^ to assist physicians in eligibility determination. These models help reduce clinical workload and improve matching precision, although human oversight remains necessary due to potential misinterpretation of ambiguous criteria. TrialGPT^9^ advances this line of work by generating trial rankings accompanied by detailed justifications, but its occasional inaccuracies highlight the importance of cautious integration into clinical workflows.

*Clinical trial planning and prediction* are increasingly supported by the integration of LLMs, which support key tasks such as trial retrieval, eligibility criteria design, site selection, and outcome forecasting. Med-monoT5^122^ has been adapted for medical passage ranking to improve the retrieval of relevant trials. CliniDigest^125^ integrates GPT-3.5 to enhance context-aware retrieval across trial documents. AutoTrial, using a two-stage GPT-2 training process, generates eligibility criteria by learning patterns from historical trials, reducing manual workload. Trial site selection has also been addressed through models such as FRAMM,^126^ which incorporate multimodal data for optimized matching and represent an early step toward LLM-driven planning. In addition, outcome prediction has been improved using LLM-based models such as HINT,^10^ SPOT,^11^ and MediTab,^12^ which outperform traditional baselines in clinical forecasting tasks.

*Document writing* in clinical settings, including discharge summaries, clinical notes, and radiology reports, has traditionally been labor intensive, but recent advances in LLMs are enabling automation across these domains. For discharge summaries, Patel et al. (2023)^127^ demonstrated ChatGPT’s potential for automatic generation, while Shing et al. (2021)^128^ employed an extractive-abstractive summarization pipeline to enhance coherence. In orthopedic documentation, Transformer-based approaches have shown higher accuracy than recurrent neural networks in generating clinical narratives.^129^ For randomized controlled trial (RCT) reports, RobotReviewer^130^ automatically extracts and summarizes key information. Recent multimodal LLMs, including MedViLL^131^ and Med-PaLM2,^132^ align visual and textual inputs to generate clinically coherent radiology reports, demonstrating the potential of language models in high-stakes diagnostic documentation.

Though existing methods improve efficiency, future clinical LLMs should aim to achieve near-perfect reliability for high-stakes decisions, seamlessly integrate multimodal data (imaging, genomics, real-world EHRs), and provide auditable rationales that align with clinical reasoning.

#### Maturity assessment

In clinical trial practice, tasks such as ICD coding, patient-trial matching, and clinical trial planning are still in the early stages of general LLM implementation. Despite limited real-world testing, the rapid development of these models, especially in processing medical knowledge, suggests a promising future for their application.^6^

### Patient results

*Patient outcome prediction* involves forecasting future clinical events using EHRs, a process increasingly supported by language models that encode clinical data to aid informed decision-making across hospital- and disease-related tasks. Hospital-related prediction tasks focus on short-term clinical events. Models such as NYUTron,^133^ fine-tuned on clinical notes, predict mortality, comorbidity, and hospital readmission. MediTab^12^ integrates diverse medical data types to improve forecasting of clinical deterioration. In contrast, disease-related tasks target long-term health outcomes, such as diagnosis, morbidity, and disease progression. MMedLlama3^134^ extends these approaches to multilingual settings by training on diverse medical corpora. MUSK^135^ combines pathology images and clinical text to improve prognosis and immunotherapy response prediction, while BiomedGPT^136^ generalizes across biomedical domains to model disease trajectories and treatment outcomes through multimodal learning. Despite these advances, LLMs face ongoing limitations in clinical decision accuracy and alignment with diagnostic guidelines,^137^ indicating the need for further refinement before widespread adoption.

Though existing methods advance predictive capabilities, future models must achieve clinical-grade reliability for critical decisions, integrate longitudinal real-world data (e.g., wearables and social determinants of health) for personalized trajectories, and provide interpretable rationales aligned with medical reasoning.

#### Maturity assessment

LLMs show promise in predicting patient outcomes, aiding in diagnosis, and prognoses. General LLMs excel at handling unstructured data from electronic medical records. For example, NYUTriton^133^ integrates with the NYU Langone Health System to predict in-hospital mortality, estimate comorbidity indices, and predict 30-day readmissions. Similarly, Google’s Med-PaLM2^132^ achieved 86.5% accuracy in medical question-answering tasks, surpassing the medical passing score, and is being tested with select client groups, including VHC Health and Mayo Clinic.

### Assistance

General-purpose LLMs such as GPT-4^13^ and Med-PaLM2^132^ play an increasing role in clinical trial support by simplifying medical content, facilitating trial-related literature retrieval, and assisting in pharmacovigilance. These models are effective in translating complex medical knowledge into patient-friendly language,^15^ thereby improving trial comprehension and engagement. Clinicians benefit from LLMs’ advanced information retrieval capabilities, enabling efficient literature searches and eligibility assessments. In pharmacovigilance, LLMs contribute by identifying drug-drug interactions^16^^,^^23^ and generating analytic code to support data processing,^13^ thus improving workflow efficiency.

#### Maturity assessment

General LLMs have advanced in clinical assistance, supporting physicians and staff in tasks like document writing. For instance, Webster et al. (2023)^138^ demonstrated their effectiveness in generating clinical notes, managing chronic condition check-ins, and summarizing patient issues. Oracle’s Clinical Digital Assistant, which handles administrative tasks via voice commands, and Google’s MedPalm2, used for information retrieval and knowledge explanation in real-world settings,^132^ further highlight the growing capabilities of these models in clinical practice.

## Future directions

LLMs have yielded impressive early successes across the drug-discovery pipeline; progress, however, remains uneven, and key limitations persist, as shown in our maturity assessment. To clarify both the challenges and the opportunities ahead, each subsection below follows a gap-first, solution-next structure: we first identify the main limitation, then propose research directions and dataset initiatives to close the gap and realize the full potential of LLMs in drug development.

### Integrating biological insights

Improving LLMs’ scientific understanding is crucial for their application in drug discovery, requiring them to grasp specialized terminologies such as SMILES and IUPAC nomenclature for molecular generation and editing. Models like GPT-4, however, have shown limitations in understanding SMILES strings.^44^ They must likewise parse EHR terminology to match trial participants. Nevertheless, effective benchmarking, a crucial first step in guiding LLM development, is hindered by the scarcity of medical question answering (QA) datasets that capture real-world tasks. Priorities include stronger biochemical explanations and DNA/RNA secondary structure reasoning. To this end, high-throughput assays such as SHAPE^139^ and DMS^140^ are providing grounding data. Advances in statistical mechanics, such as metadynamics,^141^ quantum mechanics, and molecular mechanics,^142^ are enhancing drug discovery predictions, though integration into LLMs has been slow due to interdisciplinary gaps and data compatibility challenges. With increased collaboration and improved computational resources, these techniques are expected to further enhance LLMs’ role in drug discovery.

Recent trends frame general-purpose LLMs as *tool users*: they orchestrate specialized software as human experts do, invoking external molecular dynamics analysis libraries, graph-neural encoders, or quantum packages on demand. Large-scale biological data, such as multi-nanosecond trajectories, do not need to be passed verbatim. Instead, they stream through summarization models that compress atomic motions into token-efficient latent representations (e.g., principal component projections, time-averaged contact maps, or learned spatiotemporal embeddings) before entering the LLM’s context window. While molecular dynamics trajectories exemplify this approach, the same compression and retrieval strategy applies to other voluminous datasets, including cryo-EM images, longitudinal single-cell profiles, or spatial omics atlases. Streaming memory Transformers and retrieval-augmented generation (RAG) then enable the LLM to recall arbitrary data slices without catastrophic forgetting. Such hybrid pipelines automate large-scale simulation analysis and reveal emergent biophysical patterns at wafer-scale throughput.

### Addressing ethical, privacy concerns, fairness, and misuse with LLMs

LLM-driven drug discovery faces ethical challenges: accountability, privacy, fairness, and misuse. As these models shape decisions, assigning responsibility for success or failure becomes complex. Their opaque logic and rapid evolution require updated regulation and clear ethical guidelines.

Privacy is a major concern, especially with the potential for unintended data leakage. Because LLMs can memorize training data, they may reveal sensitive multi-omics profiles. Adversaries can extract such fragments, underscoring the need for stronger safeguards. Misuse is another threat; models such as MegaSyn2 can be repurposed for harm,^143^ so regulation must block dangerous applications without stifling progress.

Fairness remains critical. Underrepresented diseases or populations in training data can lead to biased predictions that harm marginalized patients; models trained on well-studied cohorts often underperform elsewhere.

Moreover, several influential LLMs are commercial products whose weights, update cadence, and in-context training data remain opaque. License terms can restrict downstream publication or model sharing, and silent weight updates complicate scientific reproducibility. Future studies should include “model provenance” statements, analogous to data availability sections, to ensure transparency and reproducibility.^144^ Because LLM outputs arrive in fluent prose, users are prone to automation bias and may over-trust hallucinated answers compared to outputs from symbolic frameworks. Accordingly, similar to the EU AI Act,^145^ we advocate a graduated autonomy framework: low-stakes tasks (e.g., literature triage) may be fully automated; moderate-stakes tasks (e.g., hit triage) require human verification and audit logs; and high-stakes or clinical decisions demand formal uncertainty estimates, counterfactual explanations, and regulatory oversight.^146^ In the latter two cases, mandatory human-in-the-loop checkpoints are required before any clinical deployment.

While LLMs hold significant potential for advancing drug discovery, responsible development must prioritize addressing these ethical concerns to maximize benefits and minimize risks.

### Addressing hallucination

The growing use of LLMs in drug discovery presents significant challenges, particularly their tendency to “hallucinate,” producing irrelevant or incorrect responses. These errors can mislead research, wasting resources or steering efforts in unproductive directions, such as identifying incorrect biological targets or generating invalid molecular structures. In clinical settings, hallucinations may result in life-threatening implications, especially in diagnosis or data interpretation. While some biotech companies are using LLMs to interact with biological knowledge graphs to identify drug targets,^79^ these hallucinations remain a major risk. To mitigate this, strategies such as knowledge editing, parameter tuning, and plugin integration can improve factual accuracy. Grounding LLMs in retrieval-augmented generation (RAG) with external documents enhances the relevance of their outputs, while fine-tuning on debiased datasets helps reduce shortcuts and spurious correlations. Techniques like chain of thoughts prompting ensure the outputs are grounded in factual information, and refining decoding algorithms, such as factuality enhanced decoding, improves alignment with actual data, increasing the reliability of LLM-driven drug discovery.

### Improving data analysis

LLMs are increasingly applied in drug discovery to process and analyze large datasets, including numerical and spatiotemporal data, essential for understanding disease mechanisms and predicting molecular properties. While LLMs excel in text generation, they struggle with quantitative tasks like arithmetic,^13^ often producing incorrect answers due to limitations in standard tokenization methods that fail to capture the unique properties of numerical data. Recent approaches, such as digit-by-digit encoding, aim to improve numerical representation,^147^ but tasks like interpolation and extrapolation remain challenging.

Ultra-large virtual screening (ULVS), filtering $10^{8}$ to $10^{9}$ molecules, poses a related bottleneck. Autoregressive LLM scoring is typically one to three orders of magnitude slower than GPU-optimized docking, shape hashing, or graph-kernel filters. Practical pipelines can therefore adopt a cascaded approach: fast physics- or graph-based filters cull the chemical space to a few million candidates; an LLM then re-ranks this subset, leveraging its learned priors, synthesis constraints, and reasoning over assay metadata. Alternatively, each molecule can be pre-encoded into compact latent fingerprints, learned by graph autoencoders or efficient SMILES language models, and stored in a vector database; the LLM accesses these representations using RAG, thereby avoiding inefficient token-by-token inference over the entire compound library. This hybrid design amortizes computational cost while injecting biochemical intuition that purely geometric methods lack.

Additionally, LLMs struggle with spatiotemporal data, which is crucial for modeling dynamic molecular interactions. While they handle static text effectively, they face difficulties managing multidimensional, temporal data in areas such as molecular dynamics simulations and spatiotemporal transcriptomics. Improving LLMs’ spatiotemporal and multi-modal capabilities through hybrid approaches could enhance drug discovery by enabling autonomous analysis of molecular behaviors over time, thereby accelerating drug candidate identification and uncovering hidden molecular pathways.

### Multimodal-hybrid LLM ecosystems

Future progress will hinge on large language model ecosystems that are both *multimodal* and *hybrid*. Next-generation multimodal LLMs (MLLMs) will natively ingest and reason over the heterogeneous evidence base of drug discovery, including chemical graphs, protein structures, microscopy images, electronic health records, and the scientific literature, allowing experimentalists to query complex, cross-domain questions in natural language or code.^16^ At the same time, these systems must blend the complementary strengths of *general-purpose* and *specialized* models. General LLMs^4^^,^^5^ provide broad world knowledge, conversational interfaces, and chain-of-thought reasoning that help scientists frame hypotheses and interpret results. Specialized LLMs,^20^^,^^25^^,^^96^ fine-tuned on curated biochemical corpora, deliver high-fidelity predictions for niche tasks such as binding-mode rationalization, QSAR, or protein-ligand docking. A tightly coupled workflow could transform laboratory practice: for example, specialized models can execute domain-specific analyses and pass structured outputs back to a general LLM as an “orchestrator” for synthesis, explanation, and next-step planning.^148^ Such multimodal-hybrid architectures promise richer mechanistic insight, faster iteration cycles, and more reliable decision-making across the entire drug-discovery pipeline.

### Open datasets and benchmarking needs

Continued progress in AI-enabled drug discovery requires a concerted community effort to develop domain-specific benchmarks comparable to those in the general LLM field. Benchmarks such as OpenAI’s HumanEval^149^ for code generation and the MMLU^150^ benchmark for multitask language understanding have provided standardized, rapid evaluation frameworks that drive model improvements. In contrast, scientific applications still often rely on ad hoc case studies, limiting our ability to make fair comparisons or measure true progress. Recent initiatives such as ChemBench,^151^ a framework for evaluating chemical knowledge and reasoning across more than 2,700 question-answer pairs against human experts, demonstrate the power of structured, richly annotated benchmarks for the sciences. Similarly, large-scale BioNLP^152^ benchmarks for the systematic evaluation of four LLMs on twelve biomedical natural language processing (NLP) tasks offer critical insights and practical guidelines for deploying LLMs in biomedical language processing.

Future benchmarking resources in drug discovery should capture the field’s inherent multimodality by integrating chemical graphs, three-dimensional structures, high-content imaging, omics readouts, temporally resolved simulations, and mechanistic annotations. Equally important are curated synthesis corpora with atom-level provenance and cost-sustainability metadata, as well as integrative clinical knowledge graphs linking trial protocols to molecular endpoints and real-world outcomes. Publishing these assets under permissive licenses and maintaining them through community-driven crowdsourcing will establish transparent evaluation standards, seed continual pre-training, and foster the safe, reproducible deployment of large language models across the drug-discovery pipeline.

## Acknowledgments

We thank members of the Church lab for their critical reading of the manuscript and helpful discussions, including Rohit Arora, Xavier Portillo, Esther Mintzer, Juseong Lee, Katelyn Buehring, Asaf Ashkenazy Titelman, Chun-Ting Wu, Yu Wang, and Zhengkuan Tang. This work was funded by the 10.13039/501100012331LEO Foundation (LF-OC-20-000420), a grant from the 10.13039/100001934American Academy of Dermatology (AAD), and the Wyss Validation Fund. S.P. is supported by the ARC Future Fellowship (no. FT210100097) and ARC DP240101547. L.T.M. and G.I.W.’s research into artificial intelligence applications for drug discovery is supported by a 10.13039/501100000925National Health and Medical Research Council (NHMRC) of Australia Ideas grant (APP2013629). L.T.M.’s research is also supported by the National Heart Foundation of Australia (grant no. 101857), the National Health and Medical Research Council (NHMRC) of Australia, and the Department of Health and Aged Care through the Medical Research Future Fund (MRFF) Stem Cell Therapies Mission (grant no. MRF2015957).

## Author contributions

L.L., S.P., and G.C. co-supervised the project. Y.Z., H.Y.K., and J.J. conceptualized and designed the survey framework. Y.Z., H.Y.K., and J.J. conducted the literature review and analysis. Y.Z., H.Y.K., and J.J. prepared the figures and tables. M.Y. and L.T.M. provided domain expertise and contributed to the critical evaluation of surveyed methods. G.I.W. provided methodological insights. Y.Z., H.Y.K., J.J., and M.Y. drafted the manuscript. All authors reviewed, edited, and approved the final manuscript.

## Declaration of interests

G.C. has patents and interests in AI and biotech: Lila and Glotta Tech. Complete details of all relationships for G.C. can be found at http://arep.med.harvard.edu/gmc/tech.html.

## Declaration of generative AI and AI-assisted technologies in the writing process

The authors used OpenAI’s ChatGPT for proofreading, grammar checks, and stylistic edits. All core ideas, analyses, and writing were devised and authored by the research team, who take full responsibility for the manuscript’s content.
